# Light-activated nanomaterials for tumor immunotherapy

**DOI:** 10.3389/fchem.2022.1031811

**Published:** 2022-10-07

**Authors:** Fang Wang, Huijuan Duan, Weizhe Xu, Gang Sheng, Zhaogang Sun, Hongqian Chu

**Affiliations:** ^1^ Translational Medicine Center, Beijing Chest Hospital, Capital Medical University, Beijing, China; ^2^ Beijing Key Laboratory in Drug Resistant Tuberculosis Research, Beijing Tuberculosis and Thoracic Tumor Research Institute, Beijing, China

**Keywords:** antitumor immunity, light-activated, nanomaterials, immunotherapy, synergistic therapy

## Abstract

Tumor immunotherapy mainly relies on activating the immune system to achieve antitumor treatment. However, the present tumor immunotherapy used in the clinic showed low treatment efficacy with high systematic toxicity. To overcome the shortcomings of traditional drugs for immunotherapy, a series of antitumor immunotherapies based on nanomaterials have been developed to enhance the body’s antitumor immune response and reduce systematic toxicity. Due to the noninvasiveness, remote controllability, and high temporal and spatial resolution of light, photocontrolled nanomaterials irradiated by excitation light have been widely used in drug delivery and photocontrolled switching. This review aims to highlight recent advances in antitumor immunotherapy based on photocontrolled nanomaterials. We emphasized the advantages of nanocomposites for antitumor immunotherapy and highlighted the latest progress of antitumor immunotherapy based on photoactivated nanomaterials. Finally, the challenges and future prospects of light-activated nanomaterials in antitumor immunity are discussed.

## 1 Introduction

**FIGURE 1:**
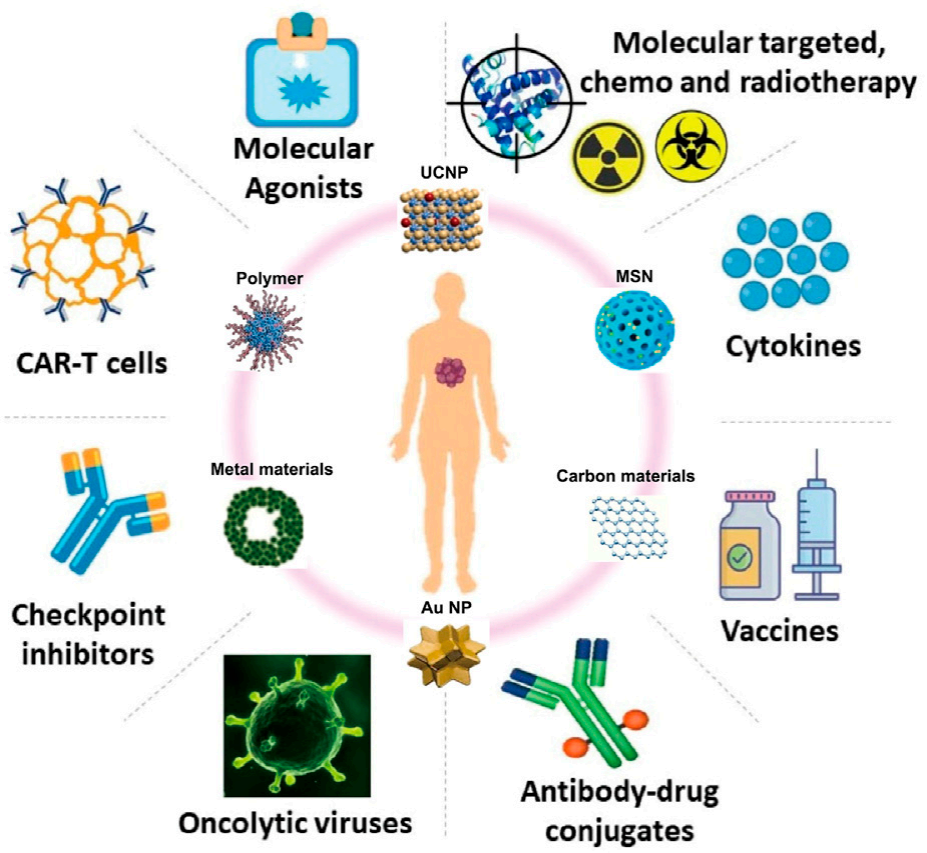
Tumor immunotherapy. (Reproduced from Chauhan et al., 2021, International Journal of Molecular Science; Guo et al., 2022, Biomaterials).

Cancer is one of the main causes of human death (Zaimy et al., 2017). Statistics show that there will be an estimated 19.3 million new cancer cases and nearly 10 million cancer deaths worldwide in 2020 (Siegel et al., 2021; Sung et al., 2021). Therefore, the exploration of early diagnosis and effective treatment methods of cancer have attracted much attention. The traditional clinical methods of tumor treatment are mainly surgery, chemotherapy and radiotherapy (Burugu et al., 2017; Hojman et al., 2018), which have defects, such as poor efficacy and high toxicity (Zeng et al., 2021). In recent years, immunotherapy has become a promising method for the treatment of malignant tumors (Riley et al., 2019; Abbott & Ustoyev, 2019; Igarashi & Sasada, 2020; Yang F. et al., 2020). Immunotherapy is the artificial enhancement or suppression of the body’s immune function in the presence of hypo- or hyperfunctioning organisms for the purpose of treating disease. Tumor immunotherapy aims to improve the overall adaptability of the immune system by modulating key immune mechanisms (Topalian, 2017) and redirecting adaptive immune cells to destroy tumor-specific targets (June et al., 2018). To date, a variety of tumor immunotherapy methods have been discovered (Figure 1) (Chauhan et al., 2021; Kumar et al., 2021; Lesch & gill, 2021; Guo et al., 2022), including immune checkpoint blockade (Ribas & Wolchok, 2018; Havel et al., 2019; He & Xu, 2020), cancer vaccines (Duong et al., 2018; DeMaria & Bilusic, 2019; Shemesh et al., 2021), cell therapy (Fry et al., 2018; Mohanty et al., 2019; Wang et al., 2020), immunomodulatory small molecules (Cukier et al., 2017; Berraondo et al., 2019; Gracia, et al., 2019), etc. However, emerging tumor immunotherapy methods still face enormous challenges, such as the low efficacy of targeted drug therapy and the inherent toxicity of immunotherapy drugs, which may lead to severe inflammatory responses and autoimmune diseases (Emens et al., 2017; Kroschinsky et al., 2017). Therefore, it is essential to find a safer and more controllable method for tumor immunotherapy.

With the rapid development of nanotechnology, the clinical application of nanomaterials is also increasing (Ulbrich et al., 2016; Zang et al., 2017; Cheng et al., 2021). Due to their special physical and chemical properties, nanoparticles have significant potential therapeutic effects in tumor immunotherapy (Velpurisiva et al., 2017; Park et al., 2018; Irvine & Dane, 2020; Muluh et al., 2021). Tumor immunotherapy mainly relies on efficient drug delivery and targeted tumor therapy. Nanomaterials are used as transport carriers to form stable nanocomplexes through encapsulation or combination, which can improve the efficacy of tumor immunotherapy and reduce drug toxicity (Gonçalves et al., 2020). Furthermore, nanomaterials can enhance the drug delivery efficiency in an active or passive manner (enhanced permeability and retention (EPR) effect) (Liu et al., 2018; Kang et al., 2020; Li et al., 2022), enabling the delivery of drugs, antibodies or other immunotherapeutic agents to preferentially accumulate at the tumor site (Jain & Stylianopoulos, 2010), which can minimize side effects and improve therapeutic efficacy (Duong et al., 2018).

To date, phototherapy has attracted extensive attention in clinical treatment due to its remote controllability, high temporal and spatial resolution, noninvasiveness and high selectivity (Li J. et al., 2019; Wang M. et al., 2019; Zhao et al., 2019). At present, light-responsive nanomaterials mainly include organic materials (photosensitizers, fluorophores and carbon-based nanoparticles) and inorganic-based nanoparticles (quantum dots, upconverting nanoparticles and gold nanoparticles) (Choi & Frangioni, 2010; Son et al., 2019). Light-activated therapy is less invasive, much more precise and safer than traditional treatments such as chemotherapy, surgery and radiation (Riley et al., 2018).

UV light is most commonly used light for photocontrolled drug delivery, release or response owing to its capability to trigger a structural change in light-responsive systems. These photochemical reaction processes then lead to nanoparticle disassembly and the subsequently controllable release of payloads. Most UV light responsive nanomaterials have been modified with photocleavable terminal groups, photocleavable side chains or multiphotocleavable linkers (Yan et al., 2013; Sun et al., 2018), and the most commonly used photocleavable protecting groups are *o*-nitrobenzyl and coumarin derivatives. According to the photochemical reaction mechanisms, light-induced structural changes are often divided into three major processes: 1) photocleavage of light-responsive units, 2) photoisomerization, and 3) photocrosslinking/-decrosslinking (Zhao et al., 2019). However, light in the ultraviolet‒visible region has poor penetration ability in biological tissues and is harmful to the skin, while near-infrared (NIR) light has deeper tissue penetration and low toxicity, so the application of NIR light to trigger tumor therapy has stronger application potential (Wang et al., 2013; Yan & Li, 2016). The mechanism of converting NIR light irradiation into UV light is discussed in the section on upconversion nanoparticles.

For immune activation triggered by light-activated nanomaterials, the strategies mainly include using light to activate cancer vaccines, chimeric antigen, receptor (CAR)-T-cell therapy, immune checkpoint blockade (ICB) therapy, cytokine therapy, and immune adjuvant therapy (Chu et al., 2021). Phototherapy can enhance the therapeutic effect by amplifying antitumor immunity, reversing the tumor immunosuppressive microenvironment (TIME) and enhancing the effect of immunotherapy by producing an extremely immunogenic tumor microenvironment (TME) (Li H. et al., 2020; Shi et al., 2020). Phototherapy can be combined with immunotherapy to eliminate metastatic tumors. Moreover, when used in combination with conventional immunotherapy, phototherapy can promote the maturation of APCs to initiate immune responses (Jiang et al., 2020).

In recent years, a variety of photocontrolled nanomaterials have been developed, such as gold nanoparticles, carbon nanomaterials, and upconversion nanoparticles (Boyer et al., 2010; Zhang et al., 2016). Here, we mainly review the application of photocontrolled nanomaterials in antitumor immunotherapy.

## 2 Light-activated nanomaterials for tumor immunotherapy

### 2.1 Polymer nanomaterial-based antitumor immunity

**FIGURE 2:**
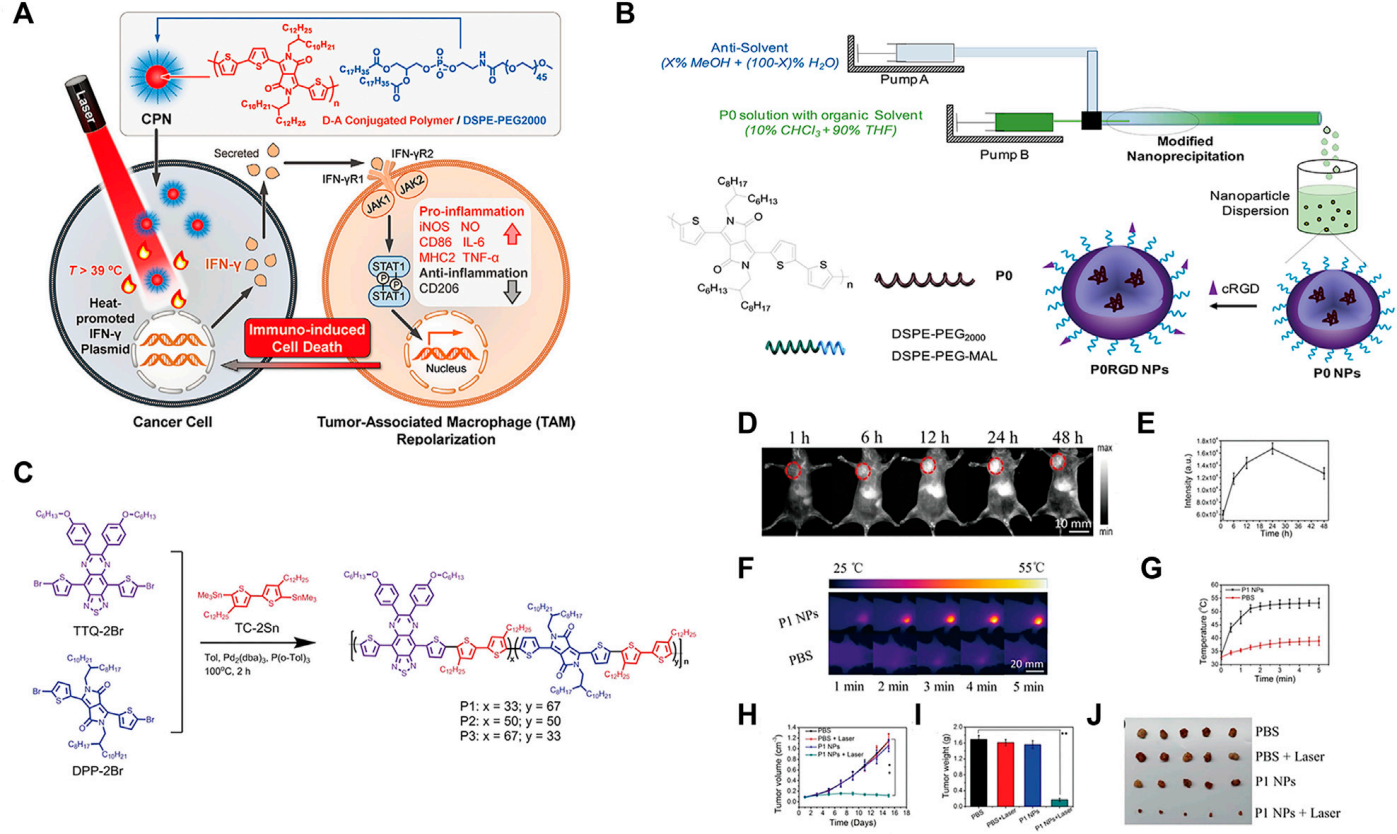
**(A)** Schematic illustration of photothermal conjugated polymer nanoparticles (CPN) for remote control cancer immunotherapy (Reproduced from Fu et al., 2021, Advance Materials). **(B)** Schematic illustration of a microfluidic glass capillary mixer for the synthesis of PORGD NPs. (Reproduced from Wang M. et al., 2019, ACS Applied Materials & Interfaces). **(C)** Synthetic route and chemical structure of three conjugated polymers. **(D)** NIR-II fluorescence images of mice with 4T1 tumors at different time points after injection with P1 NPs under 808 nm illumination. **(E)** Quantitative NIR-II fluorescence signal intensity corresponding to the tumor sites at different time points in **(D)**. **(F)** Corresponding infrared photothermal images of mice with 4T1 tumors after injection with PBS and P1 NPs under 1,064 nm laser excitation **(G)** Corresponding temperature changes at the tumor sites. **(H)** Tumor volumes and **(I)** weight growth curves of mice with 4T1 tumors treated with the four treatment groups at different time points. **(J)** Photo of excised tumors after 15 days of therapy. (Reproduced from Chen C. et al., 2021, Journal Materials Chemistry B).

Polymer-based nanoparticles can serve as excellent carriers for delivering biomolecules, drugs, genes and vaccines to tumor sites *in vivo* (Wei et al., 2021). Among them, conjugated polymers (CPs) have strong light absorption ability, good stability and biocompatibility in the NIR region. Xuan et al. reported an optogenetic system mediated by conjugated polymer nanoparticles (CPNs), which could activate immunotherapy *in situ* under NIR irradiation (Fu et al., 2021). Illumination of CPNs with NIR drives the heat shock promoter (HSP70) to trigger gene transcription of the interferon-γ (IFN-γ) cytokine. IFN-γ secreted by tumor cells induces the activation of surrounding tumor-associated macrophages through the IFN-γ-JAK-STA1 signaling pathway, which induces cancer cell killing through immunotherapy (Figure 2A).

Moreover, CPs can be facilely designed by using molecular engineering to possess certain electrical and optical properties for optimal photothermal therapy (PTT) performance (Tuncel & Demir, 2010; Guo et al., 2017; Qian et al., 2017). Wang et al. first demonstrated the synthesis of conjugated polymer nanoparticles (CP NPs) with a uniform diameter of 52 nm as PTT agents by using a modified nanoprecipitation process (Wang S. et al., 2019). Under 808 nm laser illumination, the thiolated cyclo (Arg-Gly-Asp-D-Phe-Lys (mpa)) peptide (c-RGD)-functionalized CP NPs exhibited high photothermal conversion efficiency, which activated a proinflammatory immune response and induced effective cancer cell death (Figure 2B). Furthermore, studies have found that NIR-Ⅱ light reduces light scattering and photon absorption in biological tissues, so it has better spatial resolution and lower autofluorescence intensity than traditional NIF-FI (700–900 nm) (Zhang D. X. et al., 2019; Hu et al., 2020a; Zhang et al., 2020a; Hu et al., 2020b; Yang J. et al., 2020). Thus, CPs are designed for NIR-Ⅱ because of their changeable chemical structures, adjustable NIR absorption, large Stokes shift, high extinction coefficient, and superior biocompatibility (Lin et al., 2017; Yin et al., 2017; Li T. et al., 2019; Li X. et al., 2019; Cui et al., 2020). Chen et al. designed and developed nanoparticles based on double-acceptor conjugated polymers (P1 NPs) for application in NIR-Ⅱ FI and NIR-Ⅱ PTT (Chen C. et al., 2021) (Figure 2C). The *in vivo* experiments demonstrated that P1 NPs not only exhibited high accumulation and a high sign-to background ratio (SBR) of vascular imaging at the tumor sites but also showed excellent NIR-II PTT efficiency for tumor treatment (Figures 2D–J).

Tumor cells can escape T-cell-mediated cytotoxicity using the programmed cell death protein 1 (PD-1)/programmed cell death 1 ligand 1 (PD-L1) immune checkpoint (Goodman et al., 2017), so blocking the PD-1/PD-L1 checkpoint has been extensively studied in antitumor immunity (Akinleye & Rasool, 2019). Yu et al. designed a synthetic nanoparticulated PD-L antagonist consisting of poly (ethylene glycol)-poly (lactic acid-coglycolic acid) (PEG-PLGA) nanoparticles decorated with a PD-L1 binding peptide. Nanoparticles can accumulate in the tumor site and mediate strong photothermal effects, eliminate primary tumors treated by near infrared radiation and cause strong antitumor immunity by inducing immunogenic cell death (ICD) (Yu et al., 2022).

**FIGURE 3:**
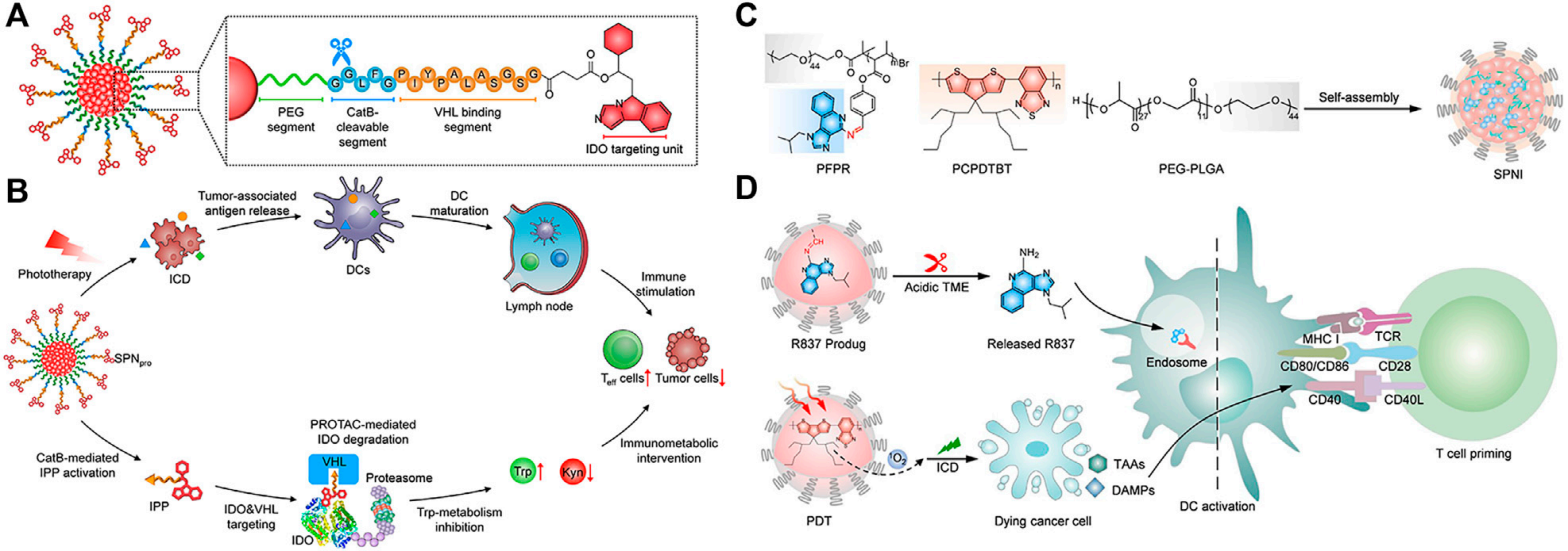
**(A)** Structure and cathepsin B (CatB)-specific activation mechanism of SPN pro. **(B)** SPN-mediated activation of two processes of photoimmunometabolic therapy. (Reproduced from Zhang et al., 2021, Nature Communitions). **(C)** Chemical structures of PFPR, PEG-PLGA, PCPDTBT, and the preparation of SPNI. **(D)** The process of immune activation mediated by SPNI-based precision photodynamic immunotherapy. (Reproduced from Liu et al., 2022, Advance Materials).

Semiconducting polymer nanoparticles (SNPs) are transformed from semiconducting polymers (SPs), which are composed of highly π-conjugated backbones (Li W. et al., 2020). Compared with most semiconductor inorganic nanoparticles, SNPs have good biocompatibility and optical properties, as well as excellent optical stability (Jiang B. P. et al., 2019; Li & Pu, 2020; Zhou et al., 2020; Zhen et al., 2021). Zhang et al. reported a semiconducting polymer nano-PROTAC (SPNpro) with phototherapy and activatable protein degrading capabilities for photoimmunometabolic cancer therapy (Zhang et al., 2021). Under NIR light irradiation, SPNpro can generate singlet oxygen to eliminate tumor cells and induce immunogenic cell death (ICD) to enhance tumor immunogenicity. In addition, cathepsin B, a cancer biomarker, can specifically activate the PROTAC of SPNpro, triggering the targeted proteolysis of immunosuppressant indoleamine 2,3-dioxygenase (IDO) in tumors. Sustained IDO degradation blocked the catabolic process of tryptophan (Trp) and promoted the activation of effector T cells (Figures 3A,B).

Liu et al. reported an amphipathic semiconductor polymer nanoimmunomodulator (SPNI) that absorbed NIR light to achieve PDT (Zhu et al., 2017) and conjugated with a Toll-like receptor 7 (TLR7) agonist (imiquimod: R837) via an acid-liable Schiff base linker (Figure 3C) (Liu et al., 2022). Introduction of R837 triggers ligation of TLR7 in endosomal membrane localization to promote DC maturation and secretion of proinflammatory cytokines (Lee et al., 2003). Under NIR light, SPNI has the photodynamic effect of direct tumor killing and death of immunogenic cancer cells. The synergistic action of the released immunogenic factor and TLR7 agonist activated by the acidic tumor microenvironment (TME) can be used as a tumor vaccine *in situ* with strong antitumor activity (Figure 3D) (Liu et al., 2022). Lyu et al. utilized the enzymatic oxidation properties of vinylidene bonds in combination with polymers to synthesize biodegradable semiconductor polymers (DPPV) and convert them into water-soluble nanoparticles (SPNV), which can enhance PA and PTT efficiencies for cancer therapy (Lyu et al., 2018). Wei et al. designed and synthesized a novel diketopyrrolopyrrole polymer nanoparticle [P(AcIID-DPP)], which exhibited strong light absorption and excellent photothermal conversion in the NIR-I to NIR-II optical region. capacity, high biocompatibility and photostability (Wei et al., 2018). In addition, nanoparticles can be efficiently absorbed by cancer cells and thermally ablated under NIR-II laser irradiation, exhibiting excellent anticancer effects. Jiang et al. synthesized an amphiphilic semiconductor polymer (PEG-PCB) that can not only be used as a diagnostic component in NIR fluorescence and PA imaging but also enable effective NIR fluorescence/PA imaging-guided photothermal therapy (Jiang et al., 2017).

Polylactic glycolic acid (PLGA) has controlled and sustained-release properties, low toxicity, and good biocompatibility and can be used for drug delivery, cancer imaging and therapy (Clawson et al., 2010; Danhier et al., 2012; Sadat Tabatabaei Mirakabad et al., 2014; Jia et al., 2018). Chen et al. discovered a kind of PLGA-IGG-R837 nanoparticle coated with the photothermal agent indocyanine green (IGG) and TLR7 ligand R837 (Chen et al., 2016). Under NIR light irradiation, PLGA-IGG-R837 ablated tumors by photothermal action and released tumor-associated antigens. Nanoparticle adjuvants loaded with R837 showed vaccine-like function, leading to immune responses. Luo et al. prepared biodegradable PLGA nanoparticles coloaded with hollow gold nanoshells (HAuNS) and anti-PD-1 peptide (APP) (AA@PN). NIR irradiation can not only trigger the release of APP and maintain a long-term immune response *in vivo* but also enable HAuNS to produce a photothermal effect to ablate tumors. The combined effect of NIR and HAuNS can produce a stronger antitumor effect (Luo et al., 2018).

### 2.2 Small molecule nanomedicine-based antitumor immunity

Small molecule nanomedicines (SMNs) refer to nanoscale drug delivery systems assembled from small molecule drugs (Ma et al., 2016; Wang Y. et al., 2017; Cheetham et al., 2017). Compared with traditional nanomedicines with complex preparation and possible toxicity of carrier materials, small molecule nanomedicines have been extensively studied (Luo et al., 2016; Li G. et al., 2021). All-drug small-molecule nanomedicines show excellent antitumor effects due to the synergistic effect of different drugs, but they have untraceable and undetermined defects (Xue et al., 2020). Adding photosensitizers to small-molecule nanomedicines can not only achieve light control but also enhance the effect of antitumor immunotherapy in combination with photodynamic therapy or photothermal therapy (Xue et al., 2019).

Li et al. self-assembled small-molecule nanoparticles by the interaction of photosensitizer ICG and epirubicin (EPI) in aqueous solution. ICG-EPI NPs exerted an excellent photothermal effect to ablate tumors under NIR laser irradiation and combined with chemotherapy drugs to further enhance the antitumor effect (Li et al., 2017). Zhang et al. assembled nanoparticles (DINP) using the hydrophobic drugs doxorubicin (DOX) and ICG and coated their surface with ruptured cancer cell membranes to form novel NIR-responsive and highly targeted small-molecule nanoparticles (DOX NPs@ICG@CCCM, DICNPs) (Zhang H. et al., 2018). The cancer cell membrane can enable DICNPs to target the tumor site. After reaching the tumor site, the cancer cell membrane was destroyed under NIR light irradiation to rapidly release DOX and ICG, thereby producing efficient chemical and photothermal effects to achieve antitumor immunotherapy. Zhang et al. assembled amphiphilic amino acids (9-fluorenylmethoxycarbonyl-L-leucine, Fmoc-ll) and photosensitive drugs (Ce6) with metal ions (Mn^2+^) to form an amino acid-porphyrin-Mn complex nanoplatform (FMCNPs) (Zhang N. et al., 2018). FMCNPs had high drug-loading capacity, good biocompatibility and MRI function and showed excellent tumor accumulation and photodynamic effects under NIR irradiation, which can effectively ablate tumors.

### 2.3 Porous silicon nanoparticle-based antitumor immunity

**FIGURE 4:**
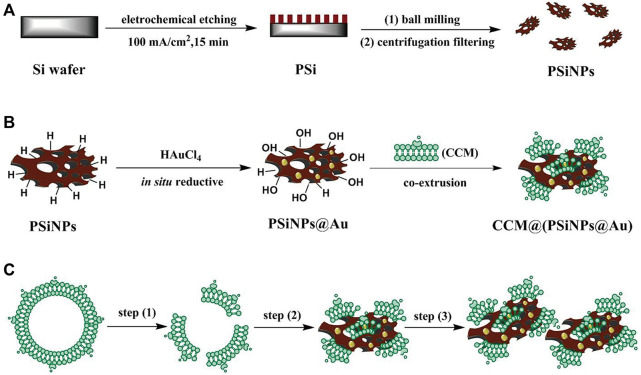
**(A–C)** The schematic simulation of **(A)** the fabrication of PSiNPs, **(B)** PSiNPs@Au and CCM@(PSiNPs@Au) samples; and **(C)** the aggregation mechanism of particles induced by CCM coating during their coextrusion. (Reproduced from Li G. et al., 2021, Advance Materials).

Due to its unique optical properties and biodegradability, porous silicon has been widely used in biomedical fields, such as drug delivery, biosensors and imaging (Martín-Palma et al., 2014; Li et al., 2018; Zhang R. et al., 2019; Tieu et al., 2021). Li et al. designed a selective photothermal and weak immunostimulatory nanovaccine based on porous silicon composite nanomaterials (Li J. et al., 2021). Porous silicon nanoparticles (PSiNPs) had a significant immunostimulatory effect on immune cells after special treatment and were coated with the cancer cell membrane (CCM) to obtain the CCM@PSiNPs@Au nanovaccine (Figure 4). Under irradiation with NIR light, immunostimulatory vaccines are released, which can induce the antitumor immune response of the body and control the overproduction of cytokines by immune cells, further enhancing the therapeutic effect of PTT.

NIR dye IR780 is a biodegradable photothermal and imaging agent that can be loaded into mesoporous silica nanoparticles (MSNs) to form biodegradable cores (Jiang et al., 2015; Zhan et al., 2017). Ma et al. coated IR780-loaded MSNs (IMs) with a prefabricated CAR-T membrane to prepare tumor-specific CAR-T membrane-wrapped nanoparticles (CIMSs) (Ma et al., 2020). Experiments *in vitro* and vivo show that CIMS has stronger tumor targeting and antitumor ability.

**FIGURE 5:**
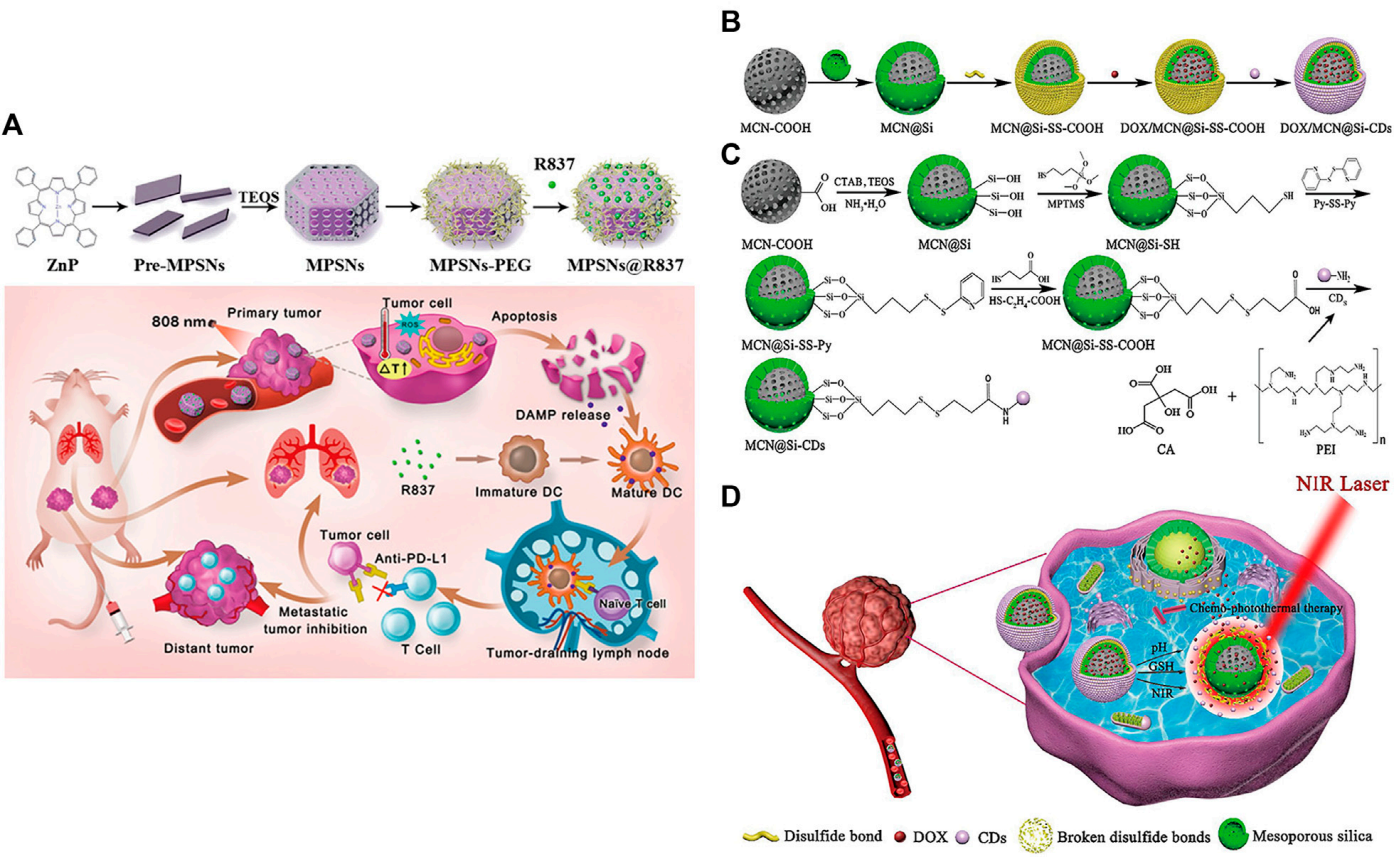
**(A)** Schematic illustration of the preparation of the core-shell zinc porphyrin nanoplatform (MPSNs@R837) and its use for synergistic antitumor immunity. (Reproduced from Yue et al., 2022, Journal of Nanobiotechnology). **(B–D)** Synthesis process of **(B)** DOX/MCN@Si-CDs and **(C)** MCN@Si-CDs. **(D)** Schematic illustration of chemo-photothermal synergistic therapy against tumors. (Reproduced from Lu et al., 2020, Colloids and Surfaces B: Biointerfaces).

MSNPs, with higher intrinsic stability, higher drug loading and larger surface area, can deliver effective concentrations of drugs to tumor sites, which provides a new research direction for targeted drug delivery (Moradipour et al., 2020; Barkat et al., 2021; Ghaferi et al., 2021; Wang et al., 2021; Sheng et al., 2022; Xie et al., 2022), such as the delivery of anti-miR therapeutics (Zhang et al., 2014; Bertucci et al., 2015; Yu et al., 2016; Khatami et al., 2021). Yue et al. developed a multifunctional nanoplatform (MPSNs@R837) formed by mesoporous hexagonal core-shell zinc porphyrin silica nanoparticles (MPSNs) loaded with R837 (Toll-like receptor 7 agonist) (Yue et al., 2022). In the presence of light sources, MPSNs@R837 can effectively destroy primary tumors through PTT and PDT. In addition, the loaded immune adjuvant R837 can be functionalized with tumor-associated antigens, promote the maturation of DCs and trigger a strong immune response (Figure 5A).

However, MSNPs suffer from low biocompatibility and dispersibility, premature drug release, and interaction with erythrocyte membranes, leading to hemolysis (Bharti et al., 2015; Zhang et al., 2017). Lu et al. constructed multistimuli-responsive mesoporous silica-coated carbon nanoparticles (DOX/MCN@Si-Cd) with high drug loading capacity and high photothermal conversion efficiency (Lu et al., 2020). The appropriate size of carbon dots (Cd) prevented the premature release of DOX (Hu et al., 2016); DOX was released rapidly at low pH and high glutathione (GSH) concentrations (Cheng et al., 2011; Cui et al., 2012; Duan et al., 2019). Local high temperature generated under NIR radiation can not only directly kill the cells but also accelerate the release of DOX and improve the sensitivity and permeability of cells. The DOX/MCN@Si-Cd compound achieved accurate drug delivery, controlled drug release and synergistic chemo-photothermal antitumor therapy (Figures 5B–D) (Lu et al., 2020).

### 2.4 Carbon nanomaterial-based antitumor immunity

Carbon nanomaterials (carbon nanotubes, carbon quantum dots, graphene oxide, carbon nanohorns (Karousis et al., 2016), etc.) are widely used in medical research due to their ideal biocompatibility, unique photothermal conversion efficiency and other physiochemical and chemical properties (Jiang Y. et al., 2019; Giordani et al., 2019; Wiehe et al., 2019; Liu et al., 2020; Lee et al., 2021; Sainz-Urruela et al., 2021). Graphene quantum dots (GQDs) have been shown to produce singlet oxygen and other ROS under specific light activation, which is the key to the phototoxicity of PDT (Ge et al., 2014). Zhang et al. proposed a hybrid photosensitizer (GQD-PEG) based on the connection of the original GQDs to polyethylene glycol (PEG), which showed significant ROS generation efficiency and excellent biocompatibility under 560 nm laser irradiation (Zhang et al., 2020b). In addition, GQD-PEG showed a strong ablative effect under irradiation and a significant increase in antitumor immune-associated cytotoxic T lymphocytes (CTLs) and proinflammatory cytokines. Liu et al. found that water-soluble C (60)(OH)(20) nanoparticles have effective antitumor activity *in vivo* and can increase the production of T helper cell type 1 (Th1) cytokines and decrease the production of Th2 cytokines (Liu et al., 2009).

**FIGURE 6:**
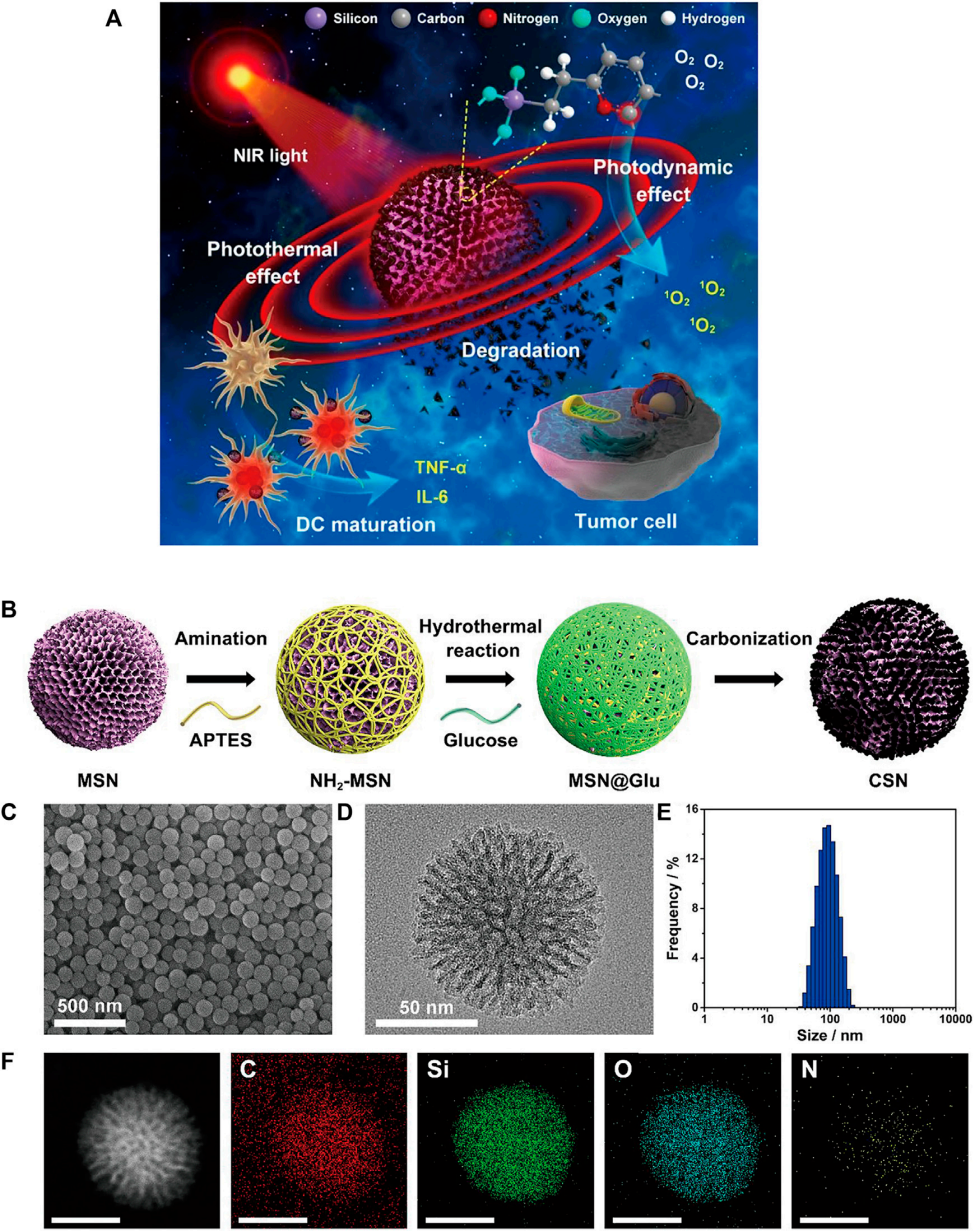
**(A)** Schematic illustration of degradable CSNs with immunoadjuvant properties for photothermal and photodynamic cancer therapy. **(B)** Schematic illustration of CSN synthesis. **(C)** SEM and **(D)** high-resolution transmission electron microscopy (HR-TEM) images of CSN. **(E)** Size distribution of CSN. **(F)** High-angle annular dark-field scanning TEM (HAADF-STEM) image and element mapping of CSN. (Reproduced from Wang et al., 2020, ACS Nano).

Single-walled carbon nanotubes (SWNTs) are characterized by strong absorbance in the NIR region (Zhou et al., 2009; Lin et al., 2022) and are able to cross cell membranes without causing cytotoxicity (Porter et al., 2007; Tajabadi, 2019). Some carbon-based nanomaterials can mature DCs and then stimulate an immune response, suggesting that they have potential immunoadjuvant properties in cancer immunotherapy (Wang et al., 2014). Zhou et al. designed a multifunctional SWNT system that can absorb NIR light to destroy tumor cells and carry immune stimulants into tumor cells to enhance tumor immunogenicity (Zhou et al., 2012). However, given the degradability of carbon nanomaterials *in vivo* (Chong et al., 2015), biodegradable carbon nanotubes or graphene oxide (GO) that have been reported thus far tend to have an inhomogeneous size or morphology, which may lead to uncertain side effects *in vivo* (Bianco, 2013). Thus, Wang et al. designed a degradable carbon-silica nanocomposite (CSN) with immunoadjuvant properties that could be degraded into small particles (∼5 nm) (Figure 6) (Wang et al., 2020). *In vivo*, the tumor inhibition efficiency of CSN was above 90% in the 4T1 tumor model and the PDX tumor model.

**FIGURE 7:**
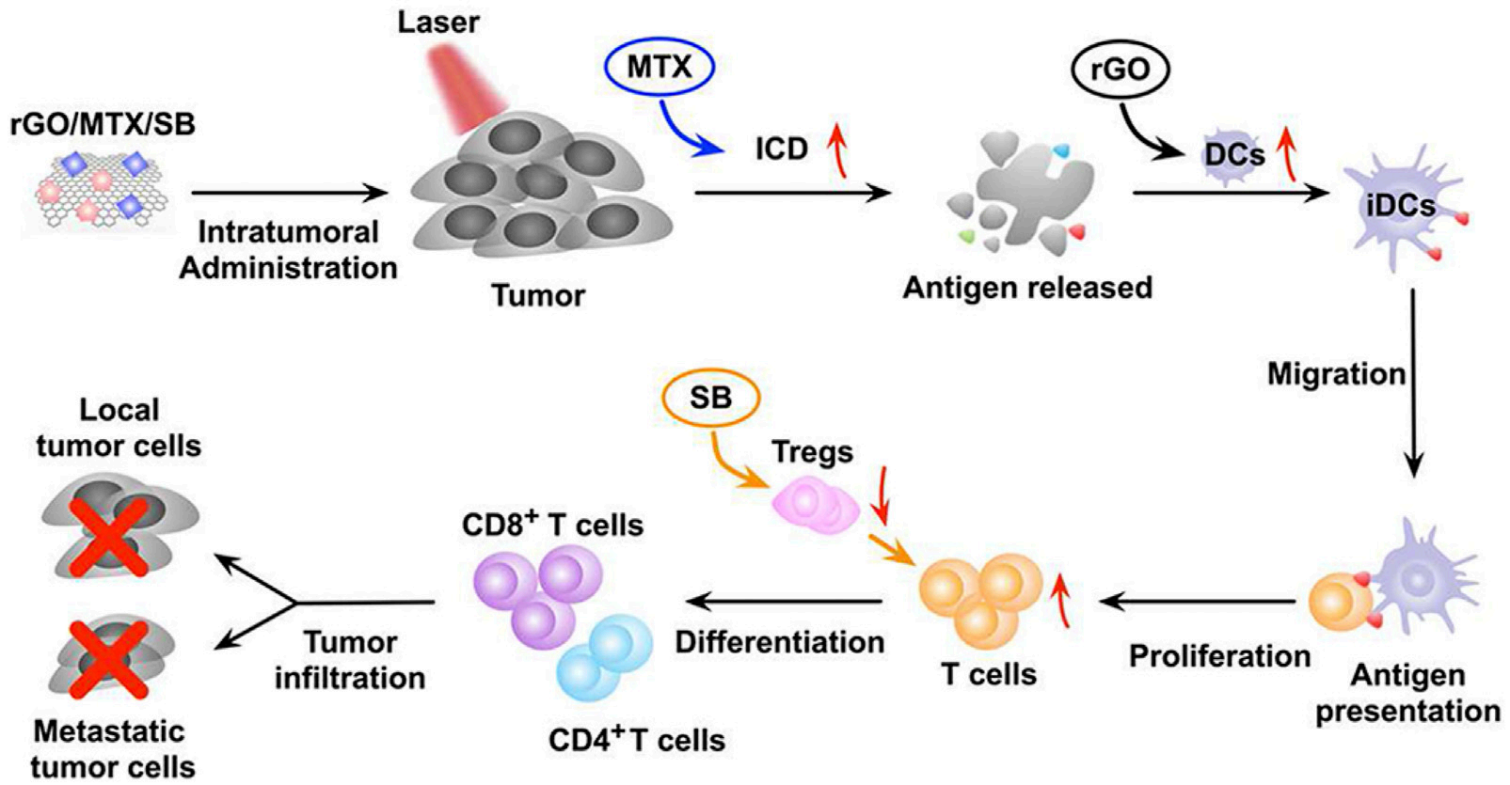
The mechanism of the antitumor immune response induced by rGO/MTX/SB-based PTT. (Reproduced from Zhou B. et al., 2021, Biomaterials).

GO is considered a promising nanomaterial for NIR drug delivery systems due to its two-dimensional film structure, biocompatibility and near infrared absorption spectroscopy (Daniyal et al., 2020). Tao et al. applied a GO-PEG-PEI nanosystem to efficiently deliver CpG, and its NIR light absorbance can control the immune stimulation activity of CpG ODNs (Tao et al., 2014). Under irradiation with NIR light, the intracellular transport of nanocarriers was accelerated due to PTT, and the immune stimulation response was significantly enhanced. Zhou et al. constructed a nanosystem (rGO/MTX/SB) that loaded the chemotherapy agent mitoxantrone (MTX) and transforming growth factor-β (TGF-β) inhibitor SB-431542 (SB) onto reduced graphene oxide (rGO) (Zhou B. et al., 2021). Under noninvasive NIR light irradiation, MTX-induced ICD effectively activated systemic antitumor immune responses, and SB helped to alter the tumor microenvironment to enhance reduced graphene oxide (rGO) (Figure 7). This synergistic therapy induced superior antitumor immunity, tumor killing and immune processes and triggered effective CTL control of metastasis.

### 2.5 Metal nanomaterial-based antitumor immunity

Metal nanomaterials have been widely used in biomedical fields due to their good physicochemical properties (Popescu, et al., 2015; Vimbela, et al., 2017). Among them, gold nanomaterials have the advantages of photocontrol ability, chemical inertness and minimal toxicity (Kohout et al., 2018; Zhou F. et al., 2021) and are widely used in the diagnosis and treatment of tumors (Singh et al., 2018; Ding et al., 2020; Guinart et al., 2020; Essawy et al., 2021). Upconversion nanoparticles are also in the category of metal nanoparticles, but they are a relatively special metal rare earth element that can be used for more effective and safer cancer treatment (Li H. et al., 2020; Liu X. et al., 2021). In addition, there are other metal nanomaterials, such as Pt, Cu and Fe, which are used in cancer therapy due to their unique physicochemical properties.

#### 2.5.1 Gold nanomaterial-based antitumor immunity

Gold nanorods (AuNRs) with tunable and strong NIR absorption are considered one of the most promising drugs for tumor therapy and diagnosis (Lee & Gaharwar, 2020). Yata et al. designed a composite immunostimulatory DNA hydrogel, mixing appropriately designed hexapods with CpG-modified gold nanoparticles to form a composite gold nanoparticle-DNA hydrogel (Yata et al., 2017). Under laser irradiation, the hydrogel released hexapods, effectively stimulating immune cells and releasing proinflammatory cytokines. Ahn et al. reported an AuNP-based therapeutic cancer vaccine carrying endogenous EDB autoantigens (Ahn et al., 2014). Gold nanoparticles can effectively deliver antigens to dendritic cells and induce antigen-specific cytotoxic T lymphocyte responses for effective cancer therapy. Khoobchandani et al. designed a novel nanodrug MGF-AuNP formed by encapsulating mangiferin (MGF) with gold nanoparticles, which can provide an effective immunomodulatory intervention by targeting the tumor microenvironment (Khoobchandani et al., 2021).

**FIGURE 8:**
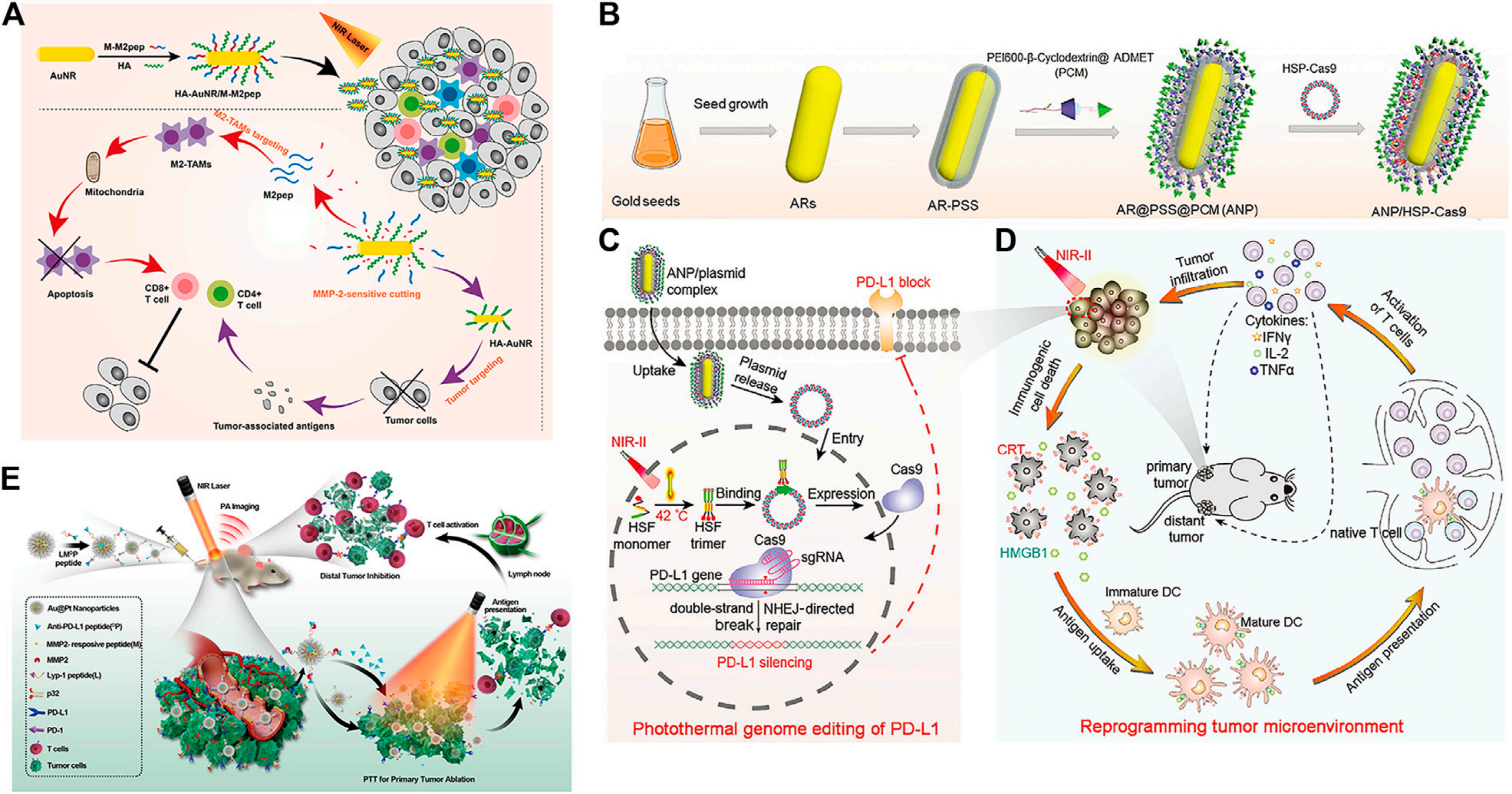
**(A)** Schematic illustration of enhanced photoimmunotherapy by the combined effect of PTT-induced immune activation and M2-TAM depletion. (Reproduced from Tian et al., 2021, Colloids and Surfaces B: Biointerfaces). **(B–D)** Schematic illustration of the photothermal genome-editing strategy for cancer immunotherapy. **(B)** Process of preparation of the ANP/HSP-Cas9 plasmid complex. **(C)** Illustration of photothermal activation for PD-L1 genome editing in tumor cells. **(D)** Photoactivable CRISPR/Cas9 strategy reprograms the immunosuppressive tumor environment. (Reproduced from Tang et al., 2021, Advance Materials). **(E)** Schematic illustration of the combination of photothermal and immunotherapy by Au@Pt-LMDP. (Reproduced from Yang et al., 2019, Journal of Controlled release).

The tumor microenvironment is an indispensable part of tumors (Pitt et al., 2016) and is one of the key factors affecting immunotherapy effects (Osipov et al., 2019; Xiao & Yu, 2021). Tian et al. designed a multifunctional nanoparticle (HA-AuNR/M-M2pep NP) to overcome the limitations of the tumor microenvironment on immunotherapy efficiency. It is composed of gold nanorods (HA-AuNR) modified with M2pep melt peptide (M-M2pep) in response to hyaluronic acid (HA) and matrix metalloproteinase-2 (MMP2). Precise PTT can be achieved under NIR light irradiation, triggering tumor immunogenic cell death and antitumor immunity irradiation (Figure 8A). Meanwhile, the nanoparticles release M2pep by cleaving MMP2-sensitive peptide, which can improve the immune activity of the TME and further enhance the antitumor efficacy (Tian et al., 2021).

Tang et al. used supramolecular gold nanorods to target and block the immune checkpoint (PD-L1-CRISPR/Cas9), which blocked the gene expression of PD-L1 under NIR light irradiation to improve the transformation of dendritic cells into T cells, promote T-cell infiltration and enhance antitumor immunity in the body (Figures 8B–D) (Tang et al., 2021). In addition, the gold nanorods can produce mild hyperthermia to induce immunogenic cell death after NIR light irradiation and further enhance tumor immunotherapy. Yang et al. reported a Au@Pt-LMDP nanosystem conjugated by Au@Pt with a reasonably designed peptide (LYP-1-PLGVRG-DPPA-1, LMDP) (Yang et al., 2019). The system can effectively eliminate primary tumors through PTT and can also act as a tumor-targeting agent activated by MMPs, releasing D-peptide antagonists of PD-L1 and stimulating the activation of cytotoxic T lymphocytes, thereby inhibiting distant tumor growth and reducing tumor metastasis (Figure 8E).

#### 2.5.2 Upconversion nanoparticle-based antitumor immunity

**FIGURE 9:**
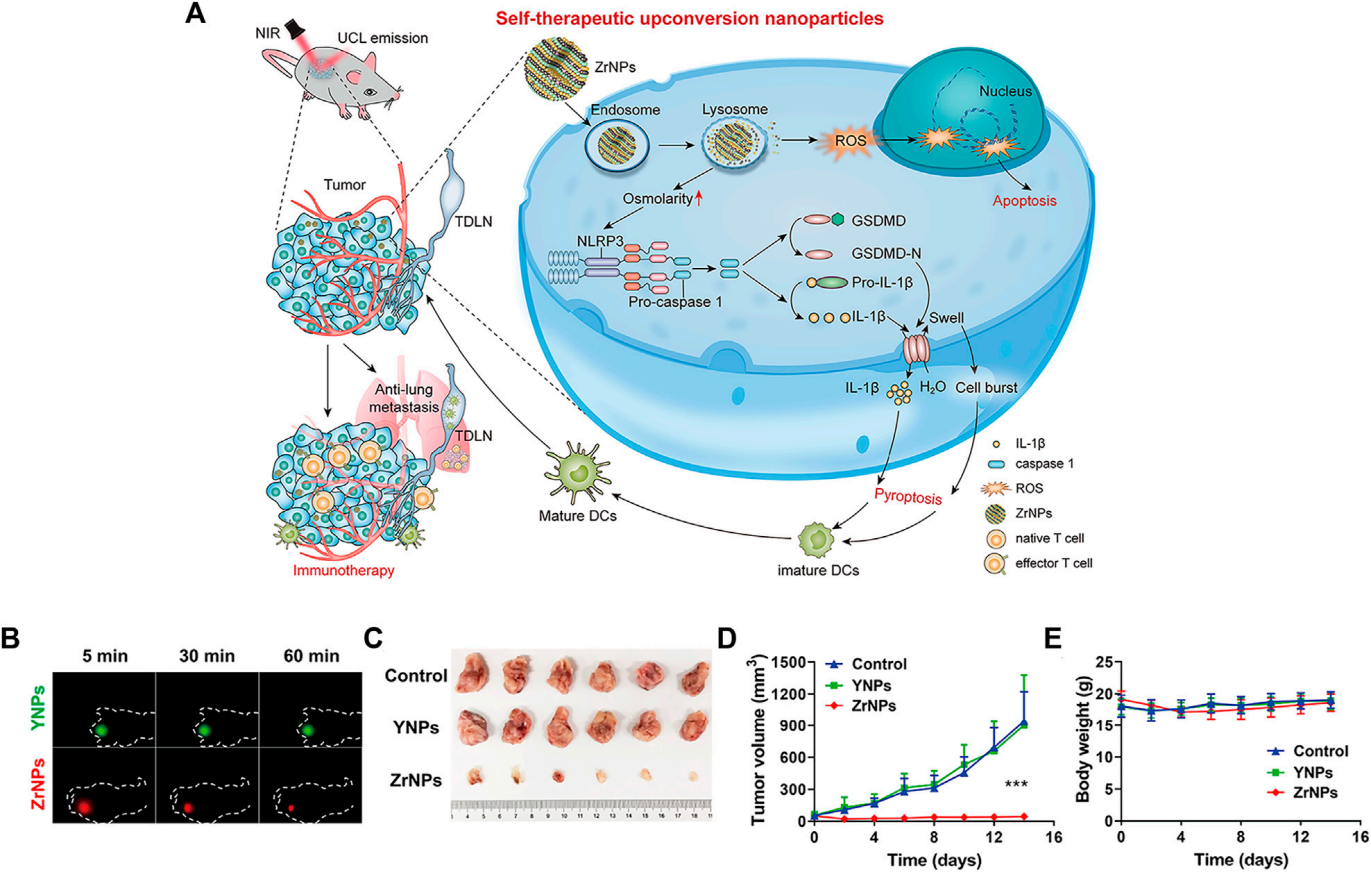
Scheme and therapeutic effects *in vivo* for antitumor therapy. **(A)** Schematic illustration of K_3_ZrF_7_:Yb/Er upconversion nanoparticles (ZrNPs) to induce pyroptosis for cancer immunotherapy. **(B)** *In vivo* UCL images of YNPs or ZrNPs at different time points. **(C)** Digital photographs of excised tumors. **(D)** Tumor growth and **(E)** body weight curves. (Reproduced from Ding et al., 2021, Nano Letters).

Upconverted nanoparticles (UCNPs) are a class of lanthanide-doped optical nanocrystals that have broad application prospects in light-controlled tumor therapy owing to their low toxicity, good chemical stability, and good photostability (Qiu et al., 2018; Wen et al., 2018). UCNPs can convert near infrared (NIR) light to UV or visible light via the sequential absorption of two or more low-energy photons, together with their deep penetrating ability, making UCNPs hot materials (Zhou et al., 2015; Wang et al., 2018; Zhou et al., 2018; Liu Y. Q. et al., 2021). Xiang et al. designed UCNPs loaded with dendritic cell (DC) vaccine antigen to label and stimulate DCs to achieve precise tracking and induce antigen-specific immune responses *in vivo*, thereby exerting antitumor immunity (Xiang et al., 2015). Our team has developed a remote-controlled antitumor immunotherapy device based on UCNPs, constructed by combining UCNPs, immunotherapeutic CpG oligonucleotides (ODN), and complementary ssDNA (PcDNA) containing a photocleavable (PC) bond (Chu et al., 2019). Under irradiation with NIR light, UCNPs can convert NIR light into high-energy UV light, which can photolytically break the PC bond and decompose PcDNA into DNA fragments, thereby releasing CpG ODNs to activate and control the body’s immune activity. Ding et al. reported biodegradable K_3_ZrF_7_:Yb/Er UCNPs (ZrNPs) as pyroptosis inducers for cancer immunotherapy (Ding et al., 2021). Sensitizer ions (Yb^3+^) absorb low-energy infrared radiation and effectively transfer excitation energy to activator ions (Er^3+^, TM^3+^, or HO^3+^), which emit high-energy ultraviolet (UV), visible, and NIR light through a multiphoton process. ZrNP-like ion banks dissolve in cancer cells and release large amounts of K+ and [ZrF_7_]^3−^ ions, further inducing an increase in oxidative pressure and reactive oxygen species (ROS). In addition, the results confirmed that ZrNPs can increase dendritic cell (DC) maturity and effector memory T-cell frequency, thereby inhibiting tumor growth and metastasis *in vivo* (Figure 9) (Ding et al., 2021).

**FIGURE 10:**
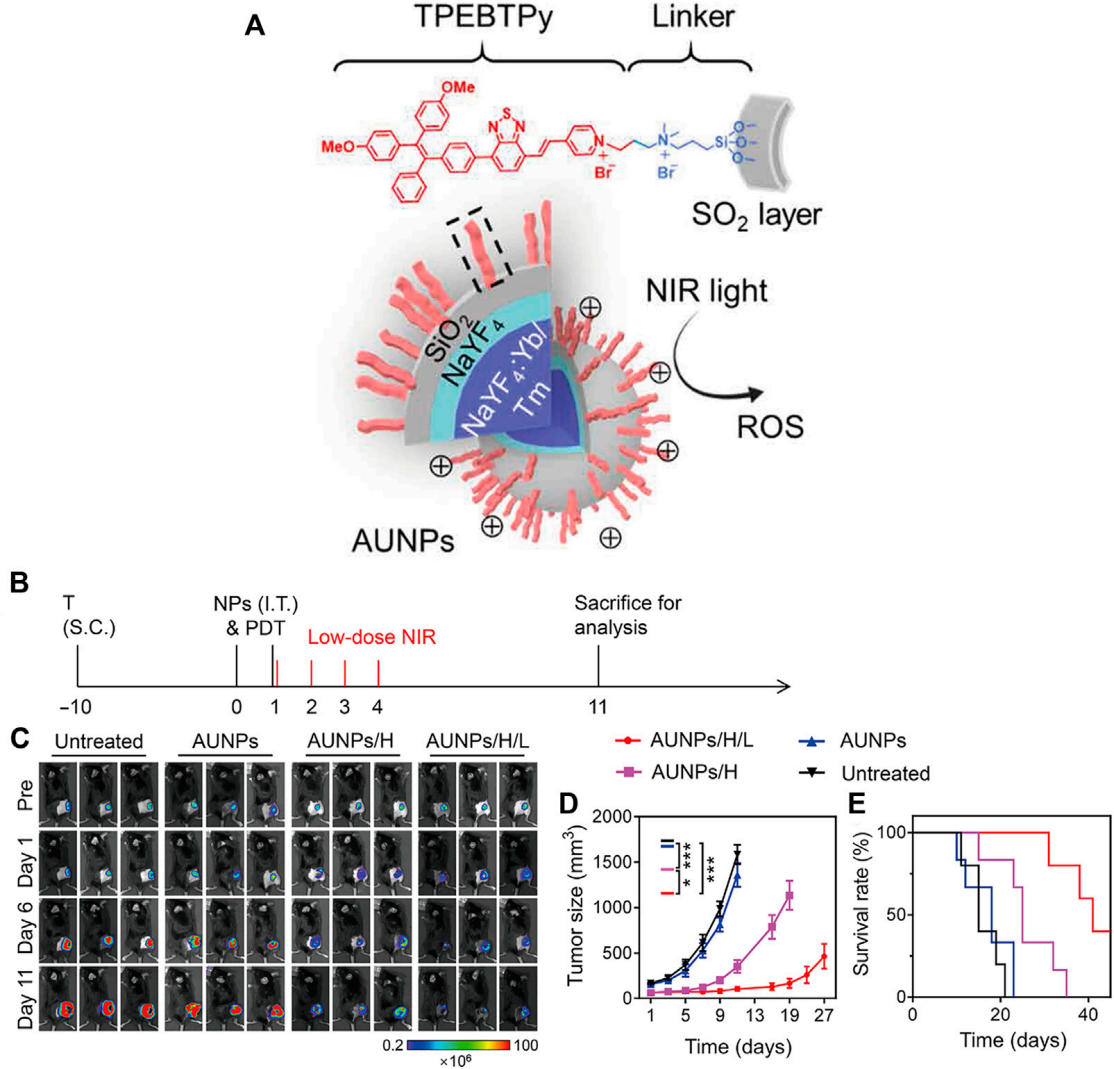
Antitumor immunotherapy with AUNP to inhibit B16F10 tumor growth. **(A)** Structure of a nanoscale immune stimulator. The dashed box indicates a linked TPEBTPy molecule on the AUNPs. **(B)** Schematic illustration of the treatment schedule. T, tumor inoculation; S.C., subcutaneous injections. **(C)** Bioluminescence images of the B16F10 tumor-bearing mice receiving different treatments. **(D)** Tumor growth curve and **(E)** survival curve of B16F10 tumor-bearing mice in the control and treated groups (*n* = 5). (Reproduced from Mao et al., 2020, Science Advances).

Mao et al. reported a nanoscale immune stimulator loaded with the aggregation-induced emission (AIE) photosensitizer TPEBTPy on UCNPs (Figure 10A) (Mao et al., 2020). TPEBTPy with AIE characteristics showed strong fluorescence and ROS generation in the aggregation state (Hu et al., 2018). The combination of TPEBTPy and UCNPs can improve light penetration and have a strong interaction. The nanomaterial enhanced the adaptive immune response to solid tumors by modulating ROS production while simultaneously activating tumor immunogenic cell death (ICD) and dendritic cells to prevent local tumor recurrence and metastasis (Figures 10B–E) (Mao et al., 2020).

**FIGURE 11:**
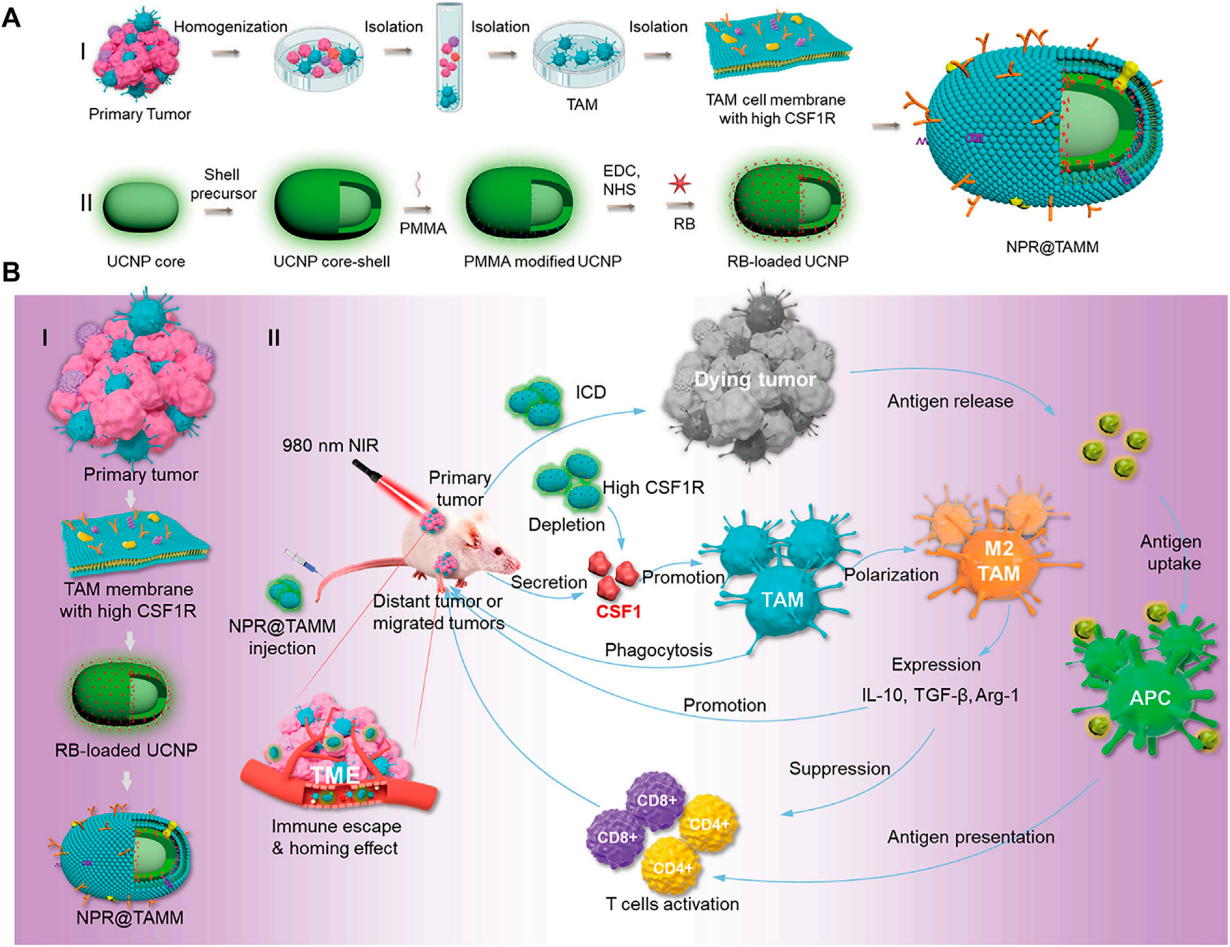
Schematic illustration of the design and mechanism of tumor-associated macrophage membrane-coated upconverting nanoparticles. **(A)** Schematic illustration of the preparation of TAMM-coated NPR@TAMMs. **(B)** Mechanism illustration of TAMM-coated NPR@TAMMs for photodynamic immunotherapy. (Reproduced from Chen Y. et al., 2021, Nano Letters).

Chen et al. reported a tumor-associated macrophage membrane (TAMM) derived from primary tumors, which was coated with a conjugated photosensitizer (NPR@TAMM) on UCNPs (Chen Y. et al., 2021). The TAMM has unique antigen-homing affinity and immune compatibility and can consume CSF1 secreted by tumor cells in the tumor microenvironment (TME), thus blocking the interaction between TAMs and cancer cells. NPR@TAMM-mediated photodynamic immunotherapy transformed macrophage activation from an immunosuppressive M2-like phenotype to a more inflammatory M1-like state and induced immunogenic cell death, thus stimulating antitumor immune efficiency by activating antigen-presenting cells (Figure 11).

**FIGURE 12:**
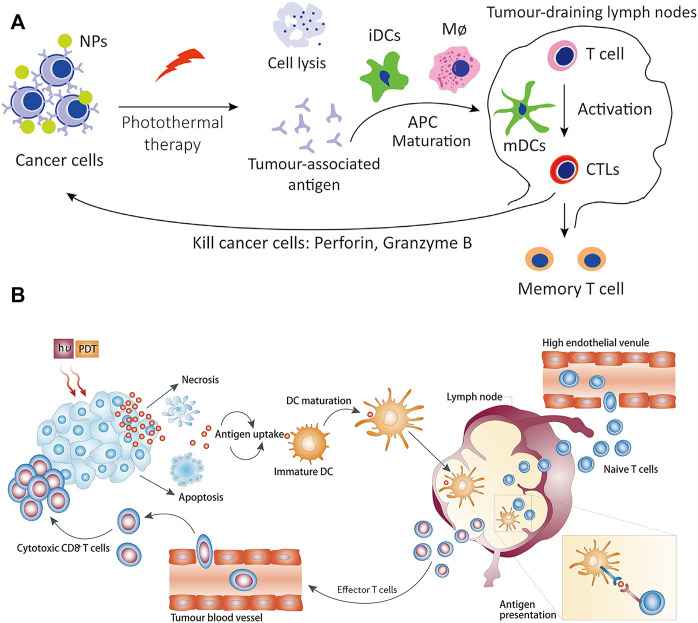
Antitumor immunotherapy based on photothermal therapy and photodynamic therapy. **(A)** Photothermal therapy increases immunogenic cell death and releases antigens that are delivered to T cells, enhancing the recognition and killing of tumor cells. (Reproduced from Li W. et al., 2020, Frontiers in Immunology). **(B)** Photodynamic therapy induces the activation of antigen-specific T cells. (Reproduced from Castano et al., 2006, Nature Reviews Cancer).

However, the therapeutic effect of single immunotherapy is still poor, and synergistic immunotherapy has a better antitumor immune effect (Sang et al., 2019; Guo et al., 2022). As shown in Figure 12A, photothermal therapy (PTT) can induce deep tissue immunogenic cell death and enhance antitumor immunotherapy (Chen et al., 2001; Chen et al., 2020; Li W. et al., 2020). Similarly, photodynamic therapy can induce immunogenic cell death and activate adaptive immune responses to tumor-associated antigens (Figure 12B) (Castano et al., 2006). Therefore, the application of synergistic immunotherapy based on light-controlled nanomaterials has more potential for clinical application.

**FIGURE 13:**
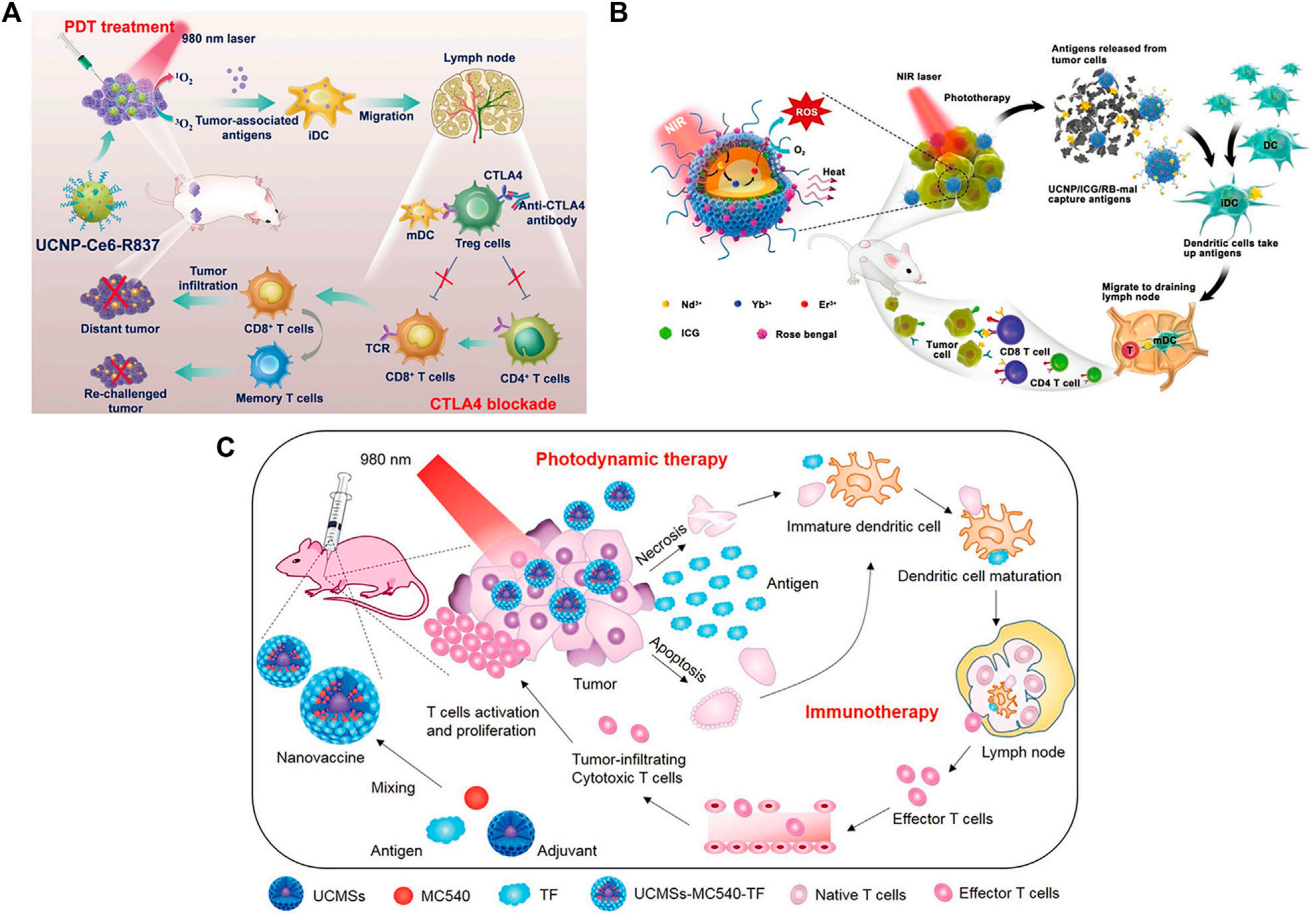
**(A)** Schematic illustration of NIR-triggered PDT with multitasking UCNPs in combination with checkpoint blockade for immunotherapy of cancer. (Reproduced from Xu et al., 2017, ACS Nano). **(B)** Schematic illustration of both the fabrication and mechanism of a near-infrared (NIR)-triggered antigen-capturing nanoplatform. (Reproduced from Wang S. et al., 2019, Advance Science). **(C)** Schematic illustration of the fabrication and mechanism of UCMSs-MC540-TF nanovaccines for PDT and immunotherapy. (Reproduced from Ding et al., 2018, Advance Materials).

Xu et al. designed a nanoplatform that combined UCNPs triggered by PDT with checkpoint blockade (Xu et al., 2017). The UCNPs were simultaneously loaded with photosensitizer e6 (Ce6) and toll-like receptor 7 agonist imiquimod (R837) to form UCNP-Ce6-R837, which was then combined with cytotoxic T lymphocyte-associated protein (CTLA-4) checkpoint blocker. The release of tumor-associated antigens through PDT under NIR irradiation also enhances antitumor immune responses with long-term immune memory function (Figure 13A). Wang et al. reported an NIR-triggered antigen nanoplatform for synergistic immunotherapy, which is a combination of lipid molecules (DSPE-PEG-mal), light absorber indocyanine green (ICG) and photosensitizer rose bengal (RB) assembled in UCNPs (Wang Z. et al., 2019). Tumor cells irradiated with NIR can release tumor-derived protein antigen (TDPA), triggering immunogenic cell death. In addition, TAPDs can be captured by the platform to induce tumor-specific immune responses (Figure 13B). Ding et al. prepared upconversion nanoparticles (UCMS) coated with mesoporous silica as an immune adjuvant for antitumor immunotherapy (Ding et al., 2018). UCMS was simultaneously loaded with the photosensitizers merocyanine 540 (MC540), chicken OVA or tumor antigens. NIR light irradiation can activate MC540, which produces ROS and releases TAA to stimulate DCs, resulting in T-cell activation and proliferation and the release of cytokines to kill tumor cells (Figure 13C).

#### 2.5.3 Other metal nanoparticle-based antitumor immunity

Zero-valent iron (ZVI) nanoparticles (NPs) have a strong reduction potential (Zou et al., 2016) and can produce a large number of reactive oxygen species (ROS) (Yang L. X. et al., 2020; He et al., 2022). Hsieh et al. utilized ZVI-NP to enhance phosphorylation-dependent ubiquitination and degradation of nuclear factor E2-related factor 2 (NRF2), resulting in excessive oxidative stress and lipid peroxidation (Hsieh et al., 2021). Furthermore, ZVI-NPs reprogrammed the polarization of tumor-associated macrophages into an antitumor M1 phenotype, increased the cytotoxic function of CD8^+^ T cells and decreased the proportion of regulatory T cells to enhance antitumor immunity. Cobalt oxide nanoparticles (CoO NPs) are promising tools for delivering antigens to antigen-presenting cells and have induced antitumor immune responses. Chattopadhyay et al. found that CoO NPs modified with N-phosphonylmethyliminodiacetic acid (PMIDA) bound lysate-promoting antigens, which are cancer antigens derived from cancer cell lysis, to form cancer cell lysate antigen-conjugated PMIDA-CoO NPs (Ag-PMIDA-CoO NPs) (Chattopadhyay et al., 2016). The nanoparticles can activate macrophages (M φ) to improve the anticancer immune response, increase serum IFN-γ and TNF-α levels and act as an adjuvant to balance proinflammatory and anti-inflammatory immune responses.

**FIGURE 14:**
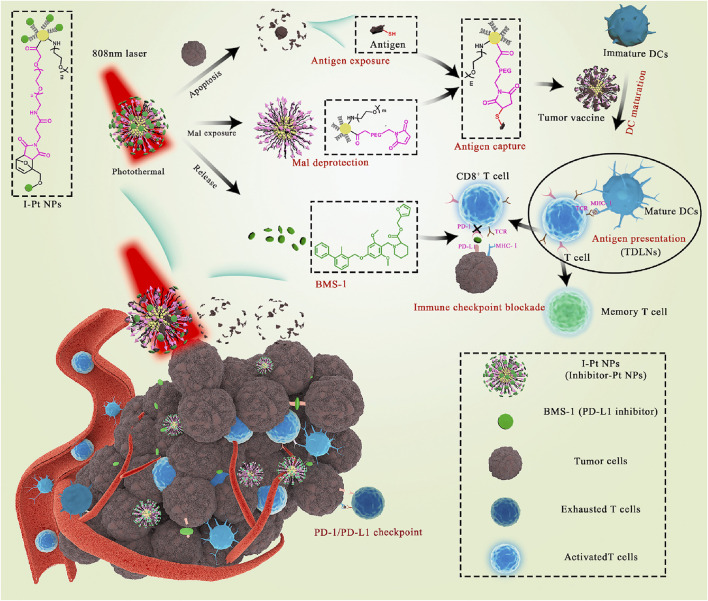
Schematic illustration of Pt NPs conjugated with BMS-1 for NIR-controlled release of inhibitor and exposure to Mal. (Reproduced from Yu et al., 2021, Bioactive Materials).

Platinum nanoparticles (Pt NPs) are selectively toxic to cancer cells (Xia et al., 2016; Shoshan et al., 2019) and enable photothermal conversion through NIR irradiation (Yang et al., 2015), leading to targeted hyperthermia (Zhou et al., 2016) and antigen release (Ma et al., 2019). Yu et al. constructed Pt NPs that conjugated PD-L1 inhibitor (BMS-1) to Mal-modified polyethylene glycol (PEG) via thermosensitive bonds (Yu et al., 2021). Under NIR irradiation, Pt NPs ablated tumors by PTT and released BMS-1 to alleviate T-cell depletion and induce effector T cells to infiltrate into tumor tissues and acted as immune adjuvants to stimulate the maturation of DCs. Mal exposed to the surface of nanoparticles captured the antigens released by tumor cells and enhanced antigen internalization and presentation (Figure 14).

**FIGURE 15:**
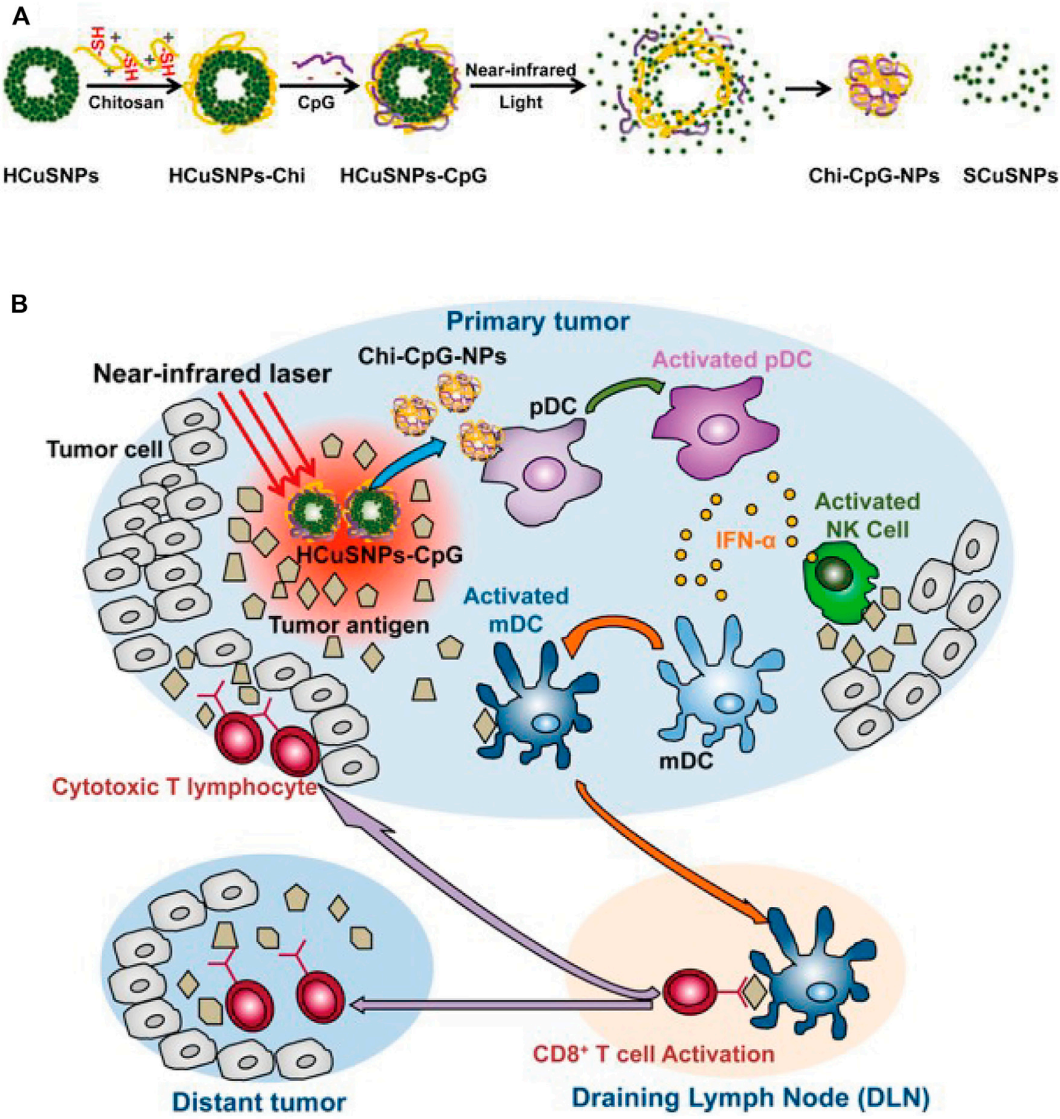
Schematic illustration of the preparation of HCuSNPs-CpG for photothermal immunotherapy. **(A)** Schematic illustration of the assembly and decomposition of the HCuSNPs-CpG conjugate. “HCuSNPs-Chi” represents chitosan-coated HCuSNPs. “Chi-CpG-NPs” represents chitosan-CpG nanocomplexes. “SCuSNPs” represents small CuS nanoparticles. **(B)** Schematic illustration of HCuSNPs-CpG-mediated photothermal immunotherapy of both primary treated and distant untreated tumors. (Reproduced from Guo et al., 2014, ACS Nano).

Hollow copper sulfide nanoparticles (HCuSNPs) are biodegradable photothermal coupling agents that can be excreted from the liver and kidney with low toxicity (Guo et al., 2013). Guo et al. reported a CuS-based transformational nano-CPG system (HCuSNPs-CpG) induced by NIR light (Guo et al., 2014). Upon NIR light irradiation, HCuSNPs-CpG structures were decomposed, reassembled and transformed into chitosan-CPG nanocomplexes, which increased the stability, tumor retention, and internalization of CpG by plasmacytoid dendritic cells and initiated effective systemic antitumor immunity by activating Toll-like receptor 9 signaling (Figure 15).

Titanium nanosheets (Ti NSs), as novel and economical two-dimensional nanomaterials, have strong NIR light absorption ability, high photothermal conversion efficiency and good biosafety (Xie et al., 2019; Yuan et al., 2022). However, Ti NSs are prone to oxidation *in vivo*, and their application in medical materials is also limited. Moreover, polyethylene glycol (PEG) can improve Ti NS stability, increase the retention time of NSs in blood circulation, and enhance the drug delivery capacity of the tumor site (Yang Y. et al., 2020).

In addition, previous studies showed that some transition-metal ions (including Fe^3+^, Cu^2+^ and Mn^2+^) can be bound to the PDA structure by coordination (Li et al., 2016; Wang Z. X. et al., 2017; Ge et al., 2017). Xu et al. prepared Fe (III) chelated PDA nanoparticles with high loading and response to release iron ions, which can improve the light absorption behavior of PDA in the NIR spectrum and endow PDA with better photothermal conversion ability (Xu et al., 2022). The *in vivo* and *in vitro* results showed that Fe-PDA could significantly inhibit tumor growth and effectively promote the repolarization of tumor-associated macrophages to the M1 mode compared with PDA. Fe-PDA combined with PTT effectively improved the efficacy of immunotherapy.

A new class of nanophotosensitizers (nPSs) based on nanoscale metal-organic frameworks (nMOFs) have attracted extensive attention in the application of PDT (Shao et al., 2020; Song et al., 2021). Lan et al. reported a novel nanophotosensitizer nanoscale metal-organic framework Fe-TBP, which can overcome tumor hypoxia and enhance the sensitivity of effective PDT, thereby initiating noninflammatory tumors for cancer immunotherapy (Lan et al., 2018). When Fe-TBP is irradiated under anoxic conditions, it can catalyze a cascade reaction to produce O_2_ through a Fenton-like reaction, and O_2_ is further converted to singlet oxygen with cytotoxicity by photoexcited porphyrins (O_2_) to produce PDT effects. In addition, the PDT-induced systemic antitumor response ameliorates α-PD-L1 ICB, leading to the regression of primary and distant tumors through a distant effect.

## 3 Future and prospects

In summary, this review discusses recent advances in light-activated nanomaterials and their applications in antitumor immunotherapy. With the progress of nanotechnology, the application of nanomaterials in antitumor immunotherapy cannot be ignored. The clinical efficiency of laser treatments is limited by the low penetration of UV, visible light or visible light and makes light-activated imaging or therapy in a dilemma. To achieve deeper tissue penetration ability, near infrared (NIR) light with low energy and long wavelength is a good choice. NIR frequency bands present an optical window for deeper penetration into biological tissue. Materials such as upconversion nanoparticles have the unique capability to efficiently convert NIR light irradiation into UV or visible light via the sequential absorption of two or more low-energy photons. This approach achieves the same goal as UV or visible light with deeper tissue penetration. Despite the described promise, there still exist challenges based on light-activated nanomaterials that need to be overcome to meet the demand in clinics. First, the toxicity of light-activated nanomaterials which is also a general concern for all nanomaterials. To date, research on light-activated nanomaterials has mainly focused on constructing new light-activated activation strategies, and the metabolism and toxicity of materials are not deeply understood. Moreover, most of the models used for nanomaterial exploration are restricted to small animals, and few studies have used large animals. Furthermore, the synthesis standards of nanomaterials, the loading content of drugs, poor solubility in the physiological environment, how to effectively preserve them, etc., as well as industry consensus, are also obstacles to the clinical application of nanomaterials. Since the mechanism of tumorigenesis varies from person to person, a single immunotherapy may not achieve satisfactory antitumor therapeutic effects, and synergistic immunotherapy is becoming an important method of antitumor therapy. It is also a great challenge to combine different therapeutic mechanisms and different materials in the same nanosystem in a rational, compatible and synergistic way to achieve efficient synergistic immunotherapy. In addition, the potential risks of photoactivated nanomaterials in clinical applications, such as systemic toxicity, complexity of clearance, and long-term effects on the human body, must also be considered. The above issues may activate future exploration in the development and improvement of light-activated nanomaterials, providing better opportunities for antitumor immunotherapy for future patients.
